# Meteorology and Epidemics of the Seventeenth Century

**Published:** 1857-04

**Authors:** J. M. Winn


### NOTES ON THE METEOROLOGY AND EPIDEMICS OF THE SEVENTEENTH CENTURY.

By J. M. WINN, M.D., F.R.CP., etc.

SCATTERED throughout Evelyn's interesting diary, which extends over a great part of the seventeenth century, and intermixed with a variety of miscellaneous matters, are numerous observations on the weather and the prevailing epidemics. In the year 1848, I was at considerable pains to collect the whole of these isolated observations, and to arrange them in some sort of order for the Royal Institution of Cornwall. That society did me the honour of printing them in its annual report. The great impulse which has lately been given to sanitary science through the SANITARY REVIEW and the continued labours of the Epidemiological Society, has induced me to bring the subject once more before the public. I have subjoined these observations of Evelyn ; and I am gratified to find that, although his remarks are not made with scientific accuracy, they are sufficiently precise to give us a general view of the climate of Britain, during a period remarkable for serious epidemics and extraordinary atmospherical changes.

I have not abbreviated Evelyn's observations, considering it better to present them in his own quaint, yet forcible style. They do not require comment; there are, however, some facts of such striking importance to be found in the Diary, that I cannot refrain from directing especial attention to them.

In 1652, a very remarkable drought of four months duration occurred. In 1675, an epidemic catarrh (influenza) appeared, after a dry summer. In 1683 and 1695, the small pox was rife, during very cold weather, in December and January. The year 1692 was remarkable for a drought in Ireland, while the weather was unusually wet in England. In 1695, very severe frosts occurred early in the month of August. It is also worthy of notice that the very severe winters of 1684 and 1685 were followed by unusually dry springs. In 1679, the autumn was wet and sickly. In 1686, storms, followed by excessive heat in June, produced sickness in the camp at Hounslow Heath. During a dry frost, in January, 1692, an unusual number of people died of apoplexy. 1693 was remarkable for being both wet and salubrious.

It may be hoped that an attentive comparison of the annexed extracts from Evelyn's *Journal*, with the more modern observations of the Royal Society, with commenced in 1773, will throw some light on the laws which govern the weather, and perhaps confirm the theory of cycles in the seasons. It would also be interesting to compare some observations by Walter Mede in the fourteenth century, the MS. copy of which is said to be deposited in the Bodleian Library.

It is deserving of remark, that, on adding together the number of years which have elapsed between the severe frost of 1685 and the severe winter of 1846, and dividing the amount by seven, there is no remainder. This fact would appear to corroborate the theory of Mr. Luke Howard, that there is a cycle of seven years in the seasons of Great Britain :—

"1652. March 25.* After a drowth of near four months,

* These extracts are taken from the second edition of EVELYN'S Memoirs, there fell so violent a tempest of hail, rain, wind, thunder and lightning, as no man had seen the like in this age, the hail being in some places four or five inches about, brake all the glass about London.

"1656. July 7. Very hot and dusty.

"1657. July 21. Fell a most prodigious rain in London, and the year was very sickly.

"1658. March 7. This had been the severest winter that any man alive had known in England. The crowes feet were frozen to their prey. Islands of ice enclosed both fish and fowl frozen, and some persons in their boats.—May 15. An epidemical sickness very mortal this spring.—June 2. An extraordinary storm of hail and rain; the season is cold as winter, the wind northerly for six months.—August 18. Strong S.W. wind (which blew down the greatest trees) all night till 3 in the morning.

"1661. September 18. An exceeding sickly and wet autumn.

"1662. January 16. There had fallen great rain without any frost or seasonable cold in England, Sweden, and the northern parts, being near as warm as midsummer in some years.—February 17. Unparalleled storm of hail, thunder, and lightning.—December 1. Thames frozen, flakes of ice encompassing our boat.

"1663. July 16. Extraordinary wet and cold season.

"1664. December 22. Exceeding cold, and a hard, long, frosty season.

"1665. January 4. Excessive sharp frost and snow.—Jan 12. Returned home, after a cold journey.—July 16. There died of the plague in London this week 1,100, and in the week following about 2,100.

"1666. January 12. The contagion, though exceedingly abated, not as yet wholly extinguished.—February 6. Contagion now almost universally ceasing.—April 15. Our parish more infected than ever, though the plague has almost ceased in London.—July 22. Our parish still infected.—July 29. The pestilence now afresh increasing in our parish.—August 26. The contagion still continuing in our parish.—September 2. The great fire in London.—September 7. The plague continuing in our parish.—October 21. This season, after so long and extraordinarie drowth in August and September, as if preparatory for the dreadful fire, was so very wet and rainy as many feared an ensuing famine.—October 28. The pestilence begun to abate in our town.

---

edited by W. Bray, Esq., Fellow of the Society of Antiquaries. London: published from the original MSS. in 1819.

"1667. March 6. Great frosts, snow, and wind, prodigious at the vernal equinox.—April 4. The cold so intense that there was hardly a leaf on a tree.

"1671. January 21. This yeare the weather was so wet, stormy, and unseasonable, as had not been known for many yeares.—September 13. A dreadful tempest.

"1672. February 3. An extraordinary snow.

"1675. October 15. I got an extreme cold, such as was afterwards so epidemical-rife over all Europe like a plague. It was after an exceeding dry summer and autumn.

"1676. December 10. There fell so deep a snow as hindered us from church.—December 12. To London, in so deep a snow as I remember not to have seen the like.—December 17. More snow falling, I was not able to get to church.

"1679. October. Very wet and sickly season.

"1680. December 12. After many daies of snow, cloudy and dark weather, the comet was very much wasted.

"1681. March 27. An extraordinary sharp cold spring, not yet a leaf on y^e trees, frost and snow lying.—April 29. But one shower of rain all this month.—May 25. There had scarce fallen any rain since Christmas.—June 12. It still continued so great a drought as had never been known in England, and it was said to be universal.

"1682. April. The season was unusually wet, with rain and thunder.

"1683. March was unusually hot and dry, and all April excessively wet.—December 23. The small pox very prevalent and mortal; the Thames frozen.—December 27. In England this year one of the severest frosts that had happened of many years.

"1684. January 1. The weather continuing tolerably severe, streets of booths were set upon the Thames; the air was so very cold and thick as for many years there had not been the like. The small pox was very mortal.—January 6. The river quite frozen.—January 9. I went cross the Thames on the ice.—January 16. Thames filled with people and tents.—January 24. Frost continuing more and more severe. Trees not only splitting as if lightning struck, but men and cattle perishing in divers places. London, by reason of the excessive coldness of the air hindering the ascent of the smoke, was so filled with the fuliginous steam of the sea-coale.—February 4. I went to Say's Court to see how the frost had dealt with my garden. The oranges and mirtills very sick, the rosemary and laurels dead to all appearance, but the cypress likely to endure it.—February 5. It began to thaw, but froze again.—February 6. The weather was set in to an absolute thaw, but the Thames still frozen.—February 10. After eight weeks missing the foraine posts, there came abundance of intelligence from abroad.—April 4. Hardly the least appearance of any spring.—July 2. There had been an excessive hot and dry spring, and such a drought continued as never was in my memorie.—July 13. Some small sprinkling of rain; the leaves dropping from the trees as in autumn.—August 10. We had now rain after such a drowth as no man in England had known.—August 24. Excessive hot; we had not above one or two considerable showers, and those storms these eight or nine months, many trees died for want of refreshment.—November 2. A sudden change from temperate warm weather to an excessive cold raine, snow, and storm, such as had seldom been known. This winter began as early and fierce as the past did late; till about Christmas there had been hardly any winter.

"1685. January 1. It prov'd so sharp weather, and so long and cruel a frost, that the Thames was frozen across; but the frost was often dissolv'd and then froze again.—June 14. Such a dearth for want of rain was never in my memory.—June 17. The exceeding drowth continues.—June 28. We had now plentiful raine, after two years excessive drowth and severe winters.—November 5. An extraordinary wett morning.—November 22. Hitherto was a very wett, warm season.—December. The winter had hitherto been extraordinary wett and mild.

"1686. January 19. So wet and mild winter had scarcely been seen in man's memory.—June 2. Such storms, raine, and foul weather, seldom known at this time of the yeare. The camp at Hounslow Heath, from sickness and other inconveniences of weather, forced to retire to quarters; the storms being succeeded by excessive hot weather, many grew sick.—June 20. An extraordinary season of violent and sudden raine.—July 13. The season very rainy.—December 29. Little appearance of any winter as yet.

"1687. May 12. This day there was such a storme of winde as had seldom happened, being a sort of hurricane. It kept the flood out of the Thames, so that people went on foote over several places above bridge: also, an earthquake in severall places in England about the time of the storme.

"1688. April 15. A dry, cold, backward spring; easterly winds.—April 29. The weather was, till now, so cold and sharp, by an almost perpetual east wind which had continued many monthes, that there was little appearance of any spring, and yet the winter was very favourable as to frost and snow.

"1689. January 7. A long frost and deepe snow; the Thames almost frozen over.—April 21. This was one of the most seasonable springs, free from the usual sharp east winds, that I have observed since the 1660.—June 23. An extraordinary drowth, to the threatening of greate wants as to the fruits of the earth.—November 10. After a very wet season, the winter came on severely.—March 17. Much wet without frost, yet the wind north and easterly.

"1690. January 11. This night there was a most extraordinary storme of wind with snow. This winter, hitherto, has been extremely wet, warm, and windy.—August 16. Unseasonable and most tempestuous weather. August 17. An extraordinary sharp, cold east wind.—November 16. Exceeding greate storms, yet a warm season.—December 20. Most of this month cold and frost.

"1691. July 26. Extraordinary hot season, yet refreshed by some thunder showers.—September 23. A great storm at sea: we lost the *Coronation* and *Harwich.*—October 14. A most pleasant autumn.—November 8 to 30. An extraordinary dry and warm season; without frost, and like a new spring, such as had not been known for many yeares.—December 26. An exceeding dry and calm winter; no rain for many months past.

"1692. January 24. A frosty and dry season continued; many persons die of apoplexy; more than usual.—February 7. An extraordinary snow fell in most parts.—April. No spring yet appearing. April 24th. Very cold and unreasonable weather, scarce a leaf on the trees.—June 9. An exceeding great storm of wind and rain. An extraordinary wet season, with greate floods.—July 25. This whole summer was exceeding wet and rainy, the like had not been known since the year 1643; *whilst in Ireland they had not known so great a drowth.*—August 14. Still an exceeding wet season.—September 15. There happened an earthquake.—October 1. This season was so exceedingly cold, by reason of a very long and tempestuous north-east wind, that this usually pleasant season was very uncomfortable. No fruit ripened kindly.

"1693. February 26. An extraordinary deep snow after almost no winter, and a sudden gentle thaw.—April 23. An extraordinary wet spring.—June 24. A very wet hay harvest, and little summer as yet.—August 1. Very lovely harvest weather, and a wholesome season; but no garden fruit.—October 31. The season continued very wet, as it had nearly all the summer (if one might call it summer), in which there was no fruit, but corn was very plentiful.—December 10. Very greate storm, with thunder and lightning.

"1694. April 24. The whole month of April without rain! —May 6. Scarcely one shower has fallen since the beginning of April. May 13th. Some refreshing showers.—June 3.

Seasonable showers ; corn and all fruits in extraordinary plenty, generally.—July. Glorious steady weather.—August 5.—Stormy and unseasonable wet weather this week.—November 22. A very sickly time, especially the small pox.

"1695. January 13. The Thames was frozen over. The deaths by small pox increased to 500 more than in the preceding week. January 20th. The frost and continual snow has now lasted nearly five weeks.—February. The long frost intermitted, but not gone.—March. The latter end of this month sharp and severe cold, with much snow and hard frost ; no appearance of spring.—April 14. After a most severe, cold, and snowy winter, without, almost, any shower for many months, the wind continuing N. and E., and not a leafe appearing ; the weather and wind now changed ; some showers fell, and there was a remission of cold. April 21st. The spring begins to appear, yet the trees hardly leafed.—July 28. A very wet season.—August 11. *The weather now so cold, that greater frosts were not always seen in the midst of winter ;* this succeeded much wet, and set harvest extremely back. July 29. Very cold weather.—December 25. Hitherto mild, dark, misty weather ; now snow and frost.

"1696. February 2. An extraordinary wet season, though temperate as to cold.—March 1. The wind continuing N. and E. all this week. March 8th. Great frost and cold.—April 12. A very fine spring season.—June 21. An exceeding rainy, cold, unseasonable summer ; yet the city was very healthy.—July 7. A northern wind altering the weather, with a continual and impetuous raine of three days and nights ; changed into perfect winter. July 12. Very unseasonable and uncertain weather.—September. Fine, seasonable weather, and a great harvest after a cold, wet summer. Scarcity in Scotland. —October 24. Unreasonable, stormy weather, and an ill seed time.—November 9. Frost began fiercely, but lasted not long. November 15 to 23. Very stormy weather ; rain and inundations.—December 13. Continuance of extreme frost and snow.

"1697. January 17. The severe frost and weather relented ; but again froze, with snow.—February 7. Severe frost continued, with snow. Souldiers in the armies and garrison towns frozen to death on their posts.—September. Very bright weather, but with sharp east wind.—October 3. So greate was the storms all this week, that neere a 1,000 people were lost going into the *Texel.*

"1698. April 21. An exceeding sharp and cold season.—May 8. An extraordinary greate snow and frost, nipping the corn and other fruits. Corn at nine shillings a bushel.—December 18. Very warm, but exceeding stormy.

"1699. February 19. A most furious wind as has not happened for many years.—March 26. After an extraordinary storm, there came up the Thames a whale, which was fifty-six feet long.—June 11. After a long drowth, we had a refreshing shower. June 25. The heat has been so great almost all this month, that I do not remember to have felt much greater in Italy, and this after a winter the wettest, though not the coldest that I remember for fifty yeares last past.—July 23. Seasonable showers after a continuance of excessive drowth and heat. —October 21. After an unusual warm and pleasant season, we were surprized with a very sharp frost.—November 24. A gentle, calm, dry, temperate weather all this season of the yeare, but now came sharp, hard frost and mist, but calm.—November 25. There happened this week so thick a mist and fog, that people lost their way in the streets.—Dec. 3. Calm, bright, and warm as in the middle of April. So continued on 21st Jan.

"1700. January 25. The weather was now altering into sharp and hard frost.—February 18. Mild and calm season, with gentle frost and little misling rain.—March 8. The season was like April for warmth and mildness. March 24. The season warm, gentle, and exceeding pleasant.—April 24. A most glorious spring, with hope of abundance of fruit of all kinds, and a propitious yeare.—June 2. A sweet season, with a mixture of refreshing showers.

"1701. January 4. An exceeding deepe snow, and melted away as suddenly. January 19. Severe frost, and such a tempest as threw down many chimnies.—May. A great dearth, no considerable raine having fallen for some months. May 17. Very plentifull showers, the wind coming west and south. —August. The weather changed from heate, not much less than in Italy or Spain for some days, to wet, dripping, and cold, with intermissions of faire.

"1703. Corn and provisions so cheap, that the farmers are unable to pay their rents.—June 13. Rains have been greate and continual, and now (neare Midsummer) cold and wet.—July 25. The last week in this month an uncommon long-continued rain, and the Sunday following thunder and lightning. —November 21. The wet and uncomfortable weather staying us from church this morning. November 26 and 27. The effects of the hurricane, and tempest of wind, rain, and lightning through all the nation, especially London, were very dismal.

"1704. This year has been very plentiful.

"1705. February 21. Remarkable fine weather. Agues and small pox much in every place.—March 11. An exceeding dry season.—June. The season very dry and hot."

